# Methyl Jasmonate Regulates Antioxidant Defense and Suppresses Arsenic Uptake in *Brassica napus* L.

**DOI:** 10.3389/fpls.2016.00468

**Published:** 2016-04-11

**Authors:** Muhammad A. Farooq, Rafaqat A. Gill, Faisal Islam, Basharat Ali, Hongbo Liu, Jianxiang Xu, Shuiping He, Weijun Zhou

**Affiliations:** ^1^Institute of Crop Science and Zhejiang Key Laboratory of Crop Germplasm, Zhejiang UniversityHangzhou, China; ^2^College of Agriculture and Food Science, Zhejiang A & F UniversityLin’an, China; ^3^Institute of Crop Science, Quzhou Academy of Agricultural SciencesQuzhou, China

**Keywords:** antioxidants, arsenic, ascorbate, glutathione, methyl jasmonate, oxidative stress, secondary metabolites

## Abstract

Methyl jasmonate (MJ) is an important plant growth regulator, involved in plant defense against abiotic stresses, however, its possible function in response to metal stress is poorly understood. In the present study, the effect of MJ on physiological and biochemical changes of the plants exposed to arsenic (As) stress were investigated in two *Brassica napus* L. cultivars (ZS 758 – a black seed type, and Zheda 622 – a yellow seed type). The As treatment at 200 μM was more phytotoxic, however, its combined application with MJ resulted in significant increase in leaf chlorophyll fluorescence, biomass production and reduced malondialdehyde content compared with As stressed plants. The application of MJ minimized the oxidative stress, as revealed via a lower level of reactive oxygen species (ROS) synthesis (H_2_O_2_ and OH^-^) in leaves and the maintenance of high redox states of glutathione and ascorbate. Enhanced enzymatic activities and gene expression of important antioxidants (*SOD*, *APX*, *CAT*, *POD*), secondary metabolites (*PAL, PPO, CAD*) and induction of lypoxygenase gene suggest that MJ plays an effective role in the regulation of multiple transcriptional pathways which were involved in oxidative stress responses. The content of As was higher in yellow seeded plants (cv. Zheda 622) as compared to black seeded plants (ZS 758). The application of MJ significantly reduced the As content in leaves and roots of both cultivars. Findings of the present study reveal that MJ improves ROS scavenging through enhanced antioxidant defense system, secondary metabolite and reduced As contents in both the cultivars.

## Introduction

Arsenic (As) pollution is an important environmental issue due to its toxicity and accumulation in irrigated areas where it causes serious threats to sustainable agriculture production (Heikens, 2006). Arsenic contamination in soil from both anthropogenic and natural resources is an increasing concern, especially in Asian countries like India and Bangladesh (Zhao et al., 2010a,b) and its consumption in the food chain poses serious threats to human health. This contaminant (As) exists in different chemical forms in which As(III) is considered more phytotoxic due to its interference with the sulfhydryl groups of enzymes and proteins. Furthermore, As is also known to induce the formation of reactive oxygen species (ROS) (Meharg and Hartley-Whitaker, 2002; Dave et al., 2013). Overproduction of ROS and subsequent oxidative stress may be the common mechanism of phytotoxicity and cause of damage to important organic constituent of plant cells (Petrov et al., 2015). To eliminate the toxic effects of ROS, plants have different enzymatic or non-enzymatic antioxidants, signaling pathways and metabolites (Piotrowska et al., 2009; Ahammed et al., 2013). However, under severe toxicity conditions, the antioxidant enzymes may not play a sufficient role in minimizing the toxic effects of heavy metal. In the recent years, the use of exogenous plant growth regulators to enhance the plant tolerance against metal stress condition has been given much attention (Bartwal et al., 2013; Li et al., 2014; Ali et al., 2015).

Methyl jasmonate (MJ), belonging to a class of cyclopentanone compounds, is a naturally and ubiquitously occurring phytohormone involved in signal transduction pathway and plant response to environmental stressors (Santino et al., 2013). The endogenous level of jasmonate increases in wounding, pathogen infection, ozone and metal stress conditions (Rao and Davis, 2001; Kanna et al., 2003; Piotrowska et al., 2009). Evidences have shown that the role of jasmonic acid (JA) is very crucial in alleviating the heavy metal effects on different plant species (Keramat et al., 2010; Yan et al., 2013). To date, studies on the role of MJ on plants growth under metal stress remain insufficient, especially at transcript level that gives a more detailed estimation of antioxidant gene function.

Oilseed rape (*Brassica napus* L.) is a member of family Brassicaceae and has been used as a potential candidate for phytoextraction (Ali et al., 2014). Nowadays, this crop is used to complete the edible oil requirements, moreover it has also been used for biofuel production (Grispen et al., 2006). Due to its higher biomass in comparison to natural metal (hyper) accumulators*, B. napus* contributes to the suitability of the environment as a phytoextraction species (Grispen et al., 2006). Plants, including *B. napus*, have different enzymatic mechanisms that jointly with other defense compounds play a crucial role in mitigating the toxic effects of heavy metal. Thus, it is of utmost importance to explore its potential against As stress under the exogenous influence of jasmonate. So, in order to obtain this objective, the effects of MJ application on physio-biochemical metabolism and molecular responses were studied in the leaves of black and yellow seeded *B. napus* exposed to As stress. A number of key components including antioxidant enzymes, ascorbate and glutathione redox states, and the expression of related genes were investigated in the present study.

## Materials and Methods

### Plant Material and Growth Conditions

The seeds of two black and yellow seeded cultivars (ZS 758 and Zheda 622) of *B. napus* (oilseed rape), in which ZS 758 is tolerant and Zheda 622 is sensitive to metal stress (Farooq et al., 2015), were obtained from College of Agriculture and Biotechnology, Zhejiang University. Seeds were treated with ethanol (70% v/v) for 3 min, and then washed three times with deionized water. Washed seeds were sown in peat moss in plastic pots (170 mm × 220 mm). Morphologically uniform seedlings at five-leaf stage were transferred into pots (five plants per pot) containing a Hoagland solution (Hoagland and Arnon, 1941). The pots were aerated with an air pump and kept in greenhouse. The solution pH was maintained at 6.0. The solution was changed after every 4 days. The light intensity was in the range of 250–350 μmol m^-2^ s^-1^, temperature was 16–20°C and the relative humidity was approximately 55–60%. After 2 weeks of acclimatization, solutions were adjusted to desired arsenic (As) concentrations (50 and 200 μM) and plants were simultaneously subjected with two concentrations of MJ (0.1 and 1 μM). The As treatment concentrations were based on findings of our previous experiment (Farooq et al., 2015). While according to earlier reports (Yan et al., 2013, 2015; Singh and Shah, 2014), different concentrations of MJ for present study were optimized in preliminary experiments, where we found that 0.1 and 1 μM of MJ showed significant tolerant effect on plants under As stress treatments. Sodium arsenite (NaAsO_2_) and MJ (C_13_H_20_O_3_) were used to maintain different concentrations of As and MJ respectively, and treatments were replicated four times. The combination of treatments were as follows: (1) control (basal nutrient); (2) 0.1 μM MJ + basal nutrient; (3) 1 μM MJ + basal nutrient; (4) 50 μM As; (5) 50 μM As + 0.1 μM MJ; (6) 50 μM As + 1 μM MJ; (7) 200 μM As; (8) 200 μM As + 0.1 μM MJ; (9) 200 μM As + 1 μM MJ.

### Morphological and Chlorophyll Fluorescence Parameters

Fourteen days after treatment, plants were harvested and separated into leaves and roots. Plant material after being harvested was placed into an oven at 80°C and weighed immediately after the removal from the oven until biomass became stable (Momoh and Zhou, 2001).

For chlorophyll fluorescence analyses, *B. napus* leaves were first dark adapted for 20 min. Chlorophyll fluorescence yield (Fv/Fm) was measured by using an imaging pulse amplitude-modulated (PAM) fluorimeter (IMAG-MAXI; Heinz Walz, Effeltrich, Germany). With an image processing software (imagewin) false color images of leaf chlorophyll fluorescence yield (Fv/Fm) data was taken. From four replications, three leaves were randomly selected of different plants from each replication. Measurement of leaves was done at five different locations and their means were calculated. Thus, for every replication, the means were calculated for 15 different locations of the three different leaves.

### Total As Concentration

For total As concentration determination, oven dried samples of shoots and roots were incinerated at 550°C for 20 h in a muffle furnace. After that, by adding 31% (m/v) HNO_3_ and 17.5% (v/v) H_2_O_2_, ash was incubated at 70°C for about 2 h. The As concentration in the digest was determined using an Atomic fluorescence spectroscopy (model AFS-230E, China).

### Endogenous JA Concentration

Endogenous JA concentration was determined by using a commercial enzyme-linked immunosorbent ELISA kit (MLBIO tech., China) according to the manufacturer instructions. About 0.1 g of plant tissue was rinsed with 1× saline phosphate-buffer (PBS) containing 137 mmol L^-1^ sodium chloride (NaCl), 2.7 mmol L^-1^ potassium chloride (KCl), 8 mmol L^-1^ disodium hydrogen phosphate (Na_2_HPO_4_), 1.46 mmol L^-1^ potassium dihydrogen phosphate (KH_2_PO_4_), then homogenized in 1 mL of 1× PBS and stored overnight at -20°C. The homogenates were centrifuged for 5 min at 10000 × *g* at 4°C after repeating the two freeze-thaw cycles and supernatant was extracted. According to the manufacturer instructions, samples and standards were added to the microtiter plate wells with HRP conjugated reagent (horseradish peroxidase), after mix gently incubated for 30 min at 37°C. Inhibition reaction takes place between JA (in standards or samples) and HRP-conjugated JA with the pre-coated antibody of JA.

### Analysis of Lipid Peroxidation (MDA) and Reactive Oxygen Species (ROS)

Lipid peroxidation was determined according to the procedure of Zhou and Leul (1999) in terms of malondialdehyde (MDA) contents in *B. napus* plant.

For measurement of hydrogen peroxide (H_2_O_2_) contents, leaf sample (0.5 g) was extracted with 0.1% (w/v) TCA (5.0 mL) in an ice bath and the extraction was centrifuged for 15 min at 12,000 *g* (Eppendorf AG, model 2231, Hamburg, Germany). The supernatant (1.5 mL) was collected after the centrifugation and mixed with 0.5 mL of 10 mM potassium phosphate buffer (pH 7.0) and 1 M KI (1 mL). The H_2_O_2_ content was calculated by using a standard curve after getting the absorbance of the samples at 390 nm (Velikova et al., 2000). For estimation of extra-cellular hydroxyl radicals (OH^-^), fresh leaf sample (0.5 g) was incubated in 1 mL of Na-phosphate buffer (10 mM) with pH 7.4 and 15 mM 2-deoxy-D-ribose at 37°C for 2 h (Halliwell et al., 1987). Following incubation, an aliquot of 0.7 mL from the above mixture were added to reaction mixture containing 3 mL of 0.5% (w/v) thiobirbuteric acid (TBA, Hi Media, Mumbai, 1% stock solution made in 5 mM NaOH) and 1 mL glacialacetic acid, heated at 100°C in a water bath for 30 min and cooled down to 41°C for 10 min before measurement the absorbance readings at 550 nm.

### Total RNA Extraction, cDNA Synthesis, and Quantitative Real-Time PCR (RT-qPCR) Assays

Total RNA was extracted from ∼100 mg of leaf and root tissues using manual (Trizol) method. Prime Script^TM^ RT reagent kit (Takara, Co. Ltd., Japan) with gDNA (genomic DNA) eraser was used to remove the genomic DNA and cDNA synthesis. cDNA samples from different treatments were assayed by quantitative real-time PCR (qRT-PCR) in the iCycler iQTM Real-time detection system (Bio-Rad, Hercules, CA, USA) by using SYBR^®^ Premix Ex Taq II (Takara, Co. Ltd., Japan).

Primers for selected genes were constructed by using the primer tools such as vector NTI with the help of sequence databases (http://www.ncbi.nlm.nih.gov). The sequences (5′ → 3′of forward (F) and reverse (R) primer of each candidate genes were presented as following;

*SOD* (F: 5′ ACGGTGTGACCACTGTGACT 3′, R: 5′ GCACCGTGTTGTTTACCATC 3′),

*POD* (F: 5′ ATGTTTCGTGCGTCTCTGTC 3′, R: 5′ TACGAGGGTCCGATCTTAGC 3′),

*CAT* (F: 5′ TCGCCATGCTGAGAAGTATC 3′, R: 5′ TCTCCAGGCTCCTTGAAGTT 3′),

*APX* (F: 5′ ATGAGGTTTGACGGTGAGC 3′, R: 5′ CAGCATGGGAGATGGTAGG 3′),

GR (F: 5′ AAGCTGGAGCTGTGAAGGTT 3′, R: 5′ AGACAGTGTTCGCAAAGCAG 3′),

*GSH* (F: 5′ TTTCCTGTTCCCTTCCAGGC 3′, R: 5′ TTCATCCGGCTGCACAACTA 3′),

*DHAR* (F: 5′ TCAGCAGCGGATTTGTCCTT 3′, R: 5′ TGCCTTGACTTGAGCGATGA 3′),

*MDHAR* (F: 5′ ACTCCCGCTCGTTTGATCTC 3′, R: 5′ CTAGCTTTGGCCACTTTCGC 3′),

*PAL* (F: 5′ GGGTTGTCGTTGACGGAGTT 3′, R: 5′ CATTATGGAGCACATCTTGG 3′)

*PPO* (F: 5′ GAATCTTGGGCTCTTTA 3′, R: 5′ TTCCATTACGGTGACTT 3′),

*CAD* (F: 5′ ATGATGTCTACACCGACGGA 3′, R: 5′ ACGTGTGGAGCAAGAAACAC 3′), *LOX* (F: 5′ TGGCCCGGCAAGTATTCATT 3′, R: 5′ CTGGTATCGTGAGGCGTACC 3′),

Actin gene (F: 5′ TTGGGATGGACCAGAAGG 3′, R: 5′ TCAGGAGCAATACGGAGC 3′)

The PCR conditions consisted of denaturation at 95°C for 3 min, followed by 40 cycles of denaturation at 95°C 30 s, annealing at 58°C for 45 s and finally extension at 72 s for 45 s. System software calculated the CT (threshold cycle) for each reaction and further mRNA quantification was performed according to the method of Livak and Schmittgen (2001). The threshold cycle (Ct) value of actin was subtracted from that of the gene of interest to obtain ΔCt value.

### Biochemical Analysis of Enzyme Activities

For enzyme activity analysis, leaf samples (0.5 g) were homogenized in 50 mM potassium phosphate buffer (pH 7.8) and centrifuged at 10,000 *g* (Eppendorf AG, model 2231, Hamburg, Germany). The supernatant was collected and further used for the analysis of the following enzyme activities. Zhang et al. (2008) method was used to determine the total superoxide dismutase (SOD, EC 1.15.1.1) activity following the inhibition of photochemical reduction due to nitro blue tetrazolium (NBT). The reaction mixture was comprised of 3 mL volume of 50 mM potassium phosphate buffer (pH 7.8), 13 mM methionine, 75 μM NBT, 2 μM riboflavin, 0.1 mM EDTA and 100 μL of enzyme extract. One unit of SOD activity was defined as the enzyme amount required to cause 50% inhibition of the NBT reduction measured at 560 nm. Catalase (CAT, EC 1.11.1.6) activity was measured according to Aebi (1984) with the use of H_2_O_2_ (extinction co-efficient 39.4 mM cm^-1^) for 1 min at A_240_ in 3 mL reaction mixture containing 50 mM potassium phosphate buffer (pH 7.0), 2 mM EDTA-Na_2_, 10 mM H_2_O_2_ and 100 μL enzyme extract. According to Zhou and Leul (1999), the activity of peroxidase (POD, EC1.11.1.7) was determined as the variation in guaiacol absorbance measured at 470 nm. The reaction solution consisted of 50 mM potassium phosphate buffer (pH 7.0), 100 μL enzyme extract, 0.4% H_2_O_2_ and 1% guaiacol.

The assay for ascorbate peroxide (APX, EC 1.11.1.11) activity was determined according to Nakano and Asada (1981) with some modification in reaction solution as 100 mM phosphate (pH 7), 0.3 mM ascorbic acid (ASA), 0.06 mM H_2_O_2_, 0.1 mM EDTA-Na_2_ and 100 μL enzyme extract. The spectrophotometric was set at 290 nm and the absorption was taken at 30 s after addition of H_2_O_2._ Following Jiang and Zhang (2002), the glutathione reductase (GR, EC 1.6.4.2) activity was determined with the oxidation of NADPH for 1 min at 340 nm (extinction coefficient 6.2 mM cm^-1^). The reaction mixture was comprised of 50 mM potassium phosphate buffer (pH 7.0), 2 mM EDTA-Na_2_, 0.15 mM NADPH, 0.5 mM GSSG and 100 μL enzyme extract in a 1 mL volume. The reaction was started by adding NADPH.

### Determination of Glutathione and Ascorbate Contents

Reduced glutathione (GSH) was analyzed according to Law et al. (1983) as leaf samples (0.5 g) were homogenized with 10% (w/v) TCA (5 mL) and centrifuged at 15,000 *g* for 15 min. For glutathione contents analysis, 150 μL supernatant was added to 100 μL of 6 mM DTNB, 50 μL of glutathione reductase (10 units mL^-1^), and 700 μL 0.3 mM NADPH. Standard curve was used to calculate the total glutathione concentration. All the reagents were prepared in 125 mM NaH_2_PO_4_ buffer, containing 6.3 mM EDTA, at pH 7.5.

The concentration of ASA was determined by a spectrophotometric assay (Łukasik et al., 2012). One gram of leaf tissue was ground in 5 mL of 5% trichloroacetic acid (TCA). The homogenate was centrifuged at 15,000 *g* for 15 min. The supernatant was used to assay ASA concentration. The reaction mixture contained 0.2 mL of plant homogenate, 0.6 mL of 0.2 M phosphate buffer pH 7.4, 1 mL of 10% TCA, 0.8 mL of 42% H_3_PO_4_, 0.8 mL of 4% α,α′-dipirydyl, and 0.4 mL of 3% FeCl_3_. The reaction mixture was incubated at 42°C for 40 min, and absorbance at 525 nm was measured against a control containing 0.2 mL of 5% TCA instead of plant homogenate after centrifugation. ASA contents were calculated from a calibration curve prepared with standard and was expressed in nmol per fresh weight.

### Determination of Secondary Metabolism-Related Enzyme Activity

Based on reaction product of cinnamic acid, the activity of phenylalanine ammonia-lyase (PAL) was assayed according to the methods of Dai et al. (2006). One unit of PAL activity was defined as the change in absorbance at A290 mL^-1^ enzyme extract. The reaction mixture for the assay contained 0.5 mL of supernatant, 2 mL of sodium borate buffer (pH 8.8) and 0.5 mL of 3 mM L-phenylalanine and incubated at 30°C for 1 h. Controls did not contain L-phenylalanine. We followed the Ruiz et al. (1999) to determine the polyphenol peroxidase (PPO) activity by monitoring the increase in the absorbance at 370 nm, where caffeic acid was used as a substrate. The assay mixture consisted of 0.9 mL sodium acetate buffer (pH 5.0), 0.1 mL catechol, and 0.1 mL enzyme extract. CAD was extracted according to the Guidi et al. (2005). Enzymatic activity was determined by measuring the increase in absorbance at 400 nm when coniferyl alcohol was oxidized to coniferaldehyde. The assay was performed for 5 min at 30°C in a total volume of 0.5 mL containing 100 mM Tris-HCl (pH 8.8), 100 mM coniferyl alcohol, 2 mM NADP and 100 mL enzyme extract.

### Statistical Analysis

The analysis of variance was computed by using the SPSS v16.0 (SPSS, Inc., USA) for statistically significant differences (*P ≤* 0.05), determined on the appropriate two-way variance analysis (ANOVA) followed by the Duncan’s multiple range tests.

## Results

### Biomass Accumulation and Total As Concentration

Results showed that higher concentration of As (200 μM) significantly decreased the plant biomass in terms of dry weight as compared to control plants (**Table 1**). However, after MJ addition in the solution, the reduction in plant biomass was alleviated, especially for the treatment of 1 μM MJ. Application of MJ to As stressed (MJ 1 + 200 μM) plants improved the dry weight of cultivars ZS 758 and Zheda 622 by 31 and 27% respectively, in shoot and 18 and 23%, respectively, in root when compared with As stressed plants (**Table 1**).

**Table 1:** Effect of different treatments of methyl jasmonate (MJ) and arsenic (As) on plant dry biomass and total arsenic (As) contents in two *Brassica napus* cultivars.

As concentration	MJ concentration	Shoot dry weight (g/plant)		Root dry weight (g/plant)		Leaf total As concentration (mg kg^-1^(d.m.)		Root total As conc. (mg kg^-1^(d.m.)	
					
(μM)	(μM)	ZS 758	Zheda 622	ZS 758	Zheda 622	ZS 758	Zheda 622	ZS 758	Zheda 622
0	0	10.58 ± 0.22ab	11.39 ± 0.32a	3.22 ± 0.014a	3.21 ± 0.015ab	0.24 ± 0.015i	0.32 ± 0.023i	0.95 ± 0.011j	1.15 ± 0.039j
	0.1	10.81 ± 0.95ab	10.75 ± 0.65ab	3.21 ± 0.012a	3.19 ± 0.005ab	0.32 ± 0.014i	0.29 ± 0.033i	1.01 ± 0.008j	0.86 ± 0.049j
	1	11.35 ± 0.35a	10.68 ± 0.65ab	3.22 ± 0.008ab	3.20 ± 0.014a	0.026 ± 0.008i	0.12 ± 0.014i	0.89 ± 0.013j	1.04 ± 0.100j
50	0	9.02 ± 0.014d	8.63 ± 0.03d	2.99 ± 0.023cd	2.74 ± 0.017e	35 ± 2.41g	40.28 ± 2.53f	937.33 ± 41.47g	1088.20 ± 7.109f
	0.1	9.51 ± 0.035bcd	8.90 ± 0.04d	3.07 ± 0.014bc	2.88 ± 0.02de	28.90 ± 2.27g	34.52 ± 2.23fg	800 ± 13.65h	991 ± 0.049g
	1	10.11 ± 0.177abc	9.14 ± 0.02cd	3.14 ± 0.011ab	3.01 ± 0.014a	20.53 ± 1.63h	31.48 ± 2.57g	694 ± 9.71i	881 ± 12.34h
200	0	4.34 ± 0.023ef	3.87 ± 0.043f	1.85 ± 0.020g	1.59 ± 0.006i	76.44 ± 1.80c	98.41 ± 1.08a	1763 ± 11.78c	2139.76 ± 33.15a
	0.1	4.68 ± 0.021ef	4.16 ± 0.012f	1.99 ± 0.029g	1.75 ± 0.024h	68.54 ± 1.13d	85.41 ± 2.78b	1625 ± 5.77d	1952.33 ± 26.85b
	1	5.65 ± 0.017e	4.95 ± 0.02ef	2.11 ± 0.008f	1.96 ± 0.025g	57.41 ± 2.11e	72.57 ± 3.81cd	1409.66 ± 8.68e	1678.66 ± 66.61d


*The data are mean ± SE from four replications and different letters within a column show significant difference at *P* ≤ 0.05 according to Duncan’s multiple range test.*

Without As stress, no significant difference among the two MJ treatments was found. The As contents was increased in both cultivars with the increase of As concentration in the nutrient solution (**Table 1**). Moreover, the higher As concentration was found in Zheda 622 than ZS 758 with roots being significantly higher than leaves, respectively. Exogenous application of MJ significantly reduced the As concentration in the leaves and roots of both *B. napus* cultivars under As stress and better effect was observed at 1 μM MJ (**Table 1**).

### Chlorophyll Fluorescence

**FIGURE 1:**
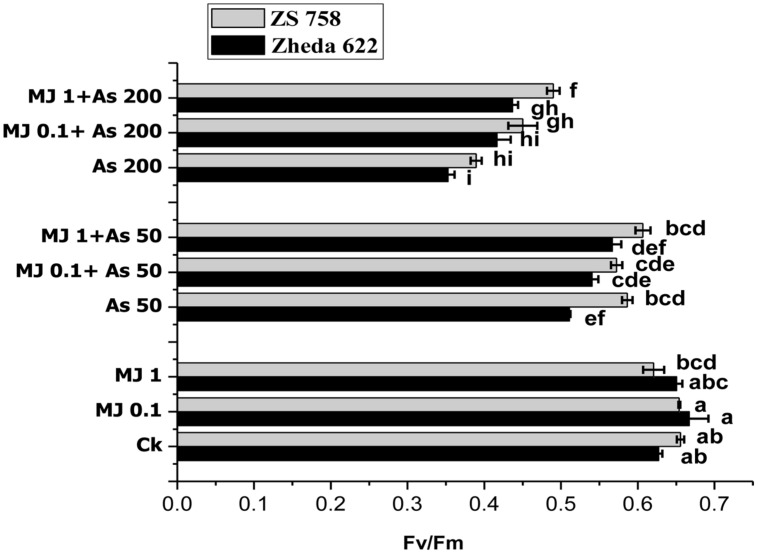
**Effect of different treatments of methyl jasmonate (MJ) and arsenic (As) on chlorophyll fluorescence in terms of photochemical efficiency of PS II (Fv/Fm) in two *Brassica napus* cultivars leaves.** Vertical bars represent the means ± SE. Different letters indicate statistically significant differences (*P ≤* 0.05) by applying Duncan’s multiple range test. The treatments consist of the control, two concentrations of MJ and As levels, and their combinations.

Lower As treatment (50 μM) in cultivar ZS 758 did not show any significant difference in chlorophyll fluorescence yield (Fv/Fm), while cultivar Zheda 622 showed significant decrease in Fv/Fm ratio, compared to their respective control (**Figure 1**). Under higher As concentration (200 μM), Fv/Fm ratio was significant decrease in both *B. napus* cultivars. When compared with control plants, the decrease in Fv/Fm ratio was 44% in cultivar Zheda 622 followed by ZS 758 (40%). In contrast, the exogenous MJ application under As stress enhanced the chlorophyll fluorescence yield (Fv/Fm) significantly as compared with As treated plants. The most significant improvement contributed by MJ was observed at 1 μM concentration. At this treatment (1 μM MJ + 200 μM As), the Fv/Fm ratio was increased by 26% in cultivar ZS 758 and 23% in Zheda 622, respectively, as compared to higher As (200 μM) treatment (**Figure 1**).

### Lipid Peroxidation and ROS Accumulation

**FIGURE 2:**
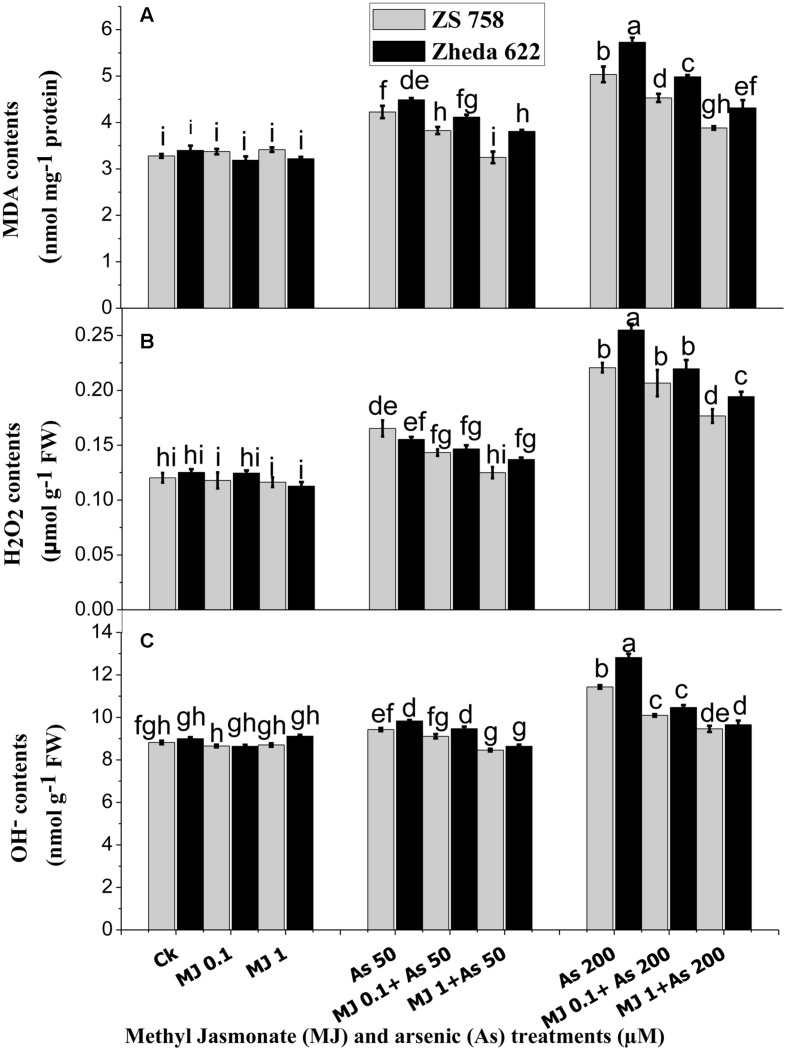
**Effect of different treatments of MJ and arsenic (As) on oxidative stress in terms of **(A)** malondialdehyde (MDA) and ROS such as **(B)** hydrogen peroxide (H_2_O_2_) and **(C)** hydroxyl radicals (OH^-^) contents in two *B. napus* cultivars.** Vertical bars represent the mean ± SE. Different letters indicate statistically significant differences (*P* ≤ 0.05) by applying Duncan’s multiple range test.

In order to evaluate the As-induced oxidative stress, the contents of H_2_O_2_ and OH^-^, representing the ROS and MDA contents are presented in **Figure 2.** At higher concentration (200 μM) of As, MDA production was significantly increased in the leaves of both cultivars as compared with the lower level of As stress (50 μM) and control plants (**Figure 2A**). At higher As (200 μM) stress, MDA content increased 62% in ZS 758 and 75% in Zheda 622, as compared with untreated control plants. However, exogenously applied MJ significantly reduced the As-induced MDA formation in the leaves of both cultivars. As shown in **Figure 2A**, the alleviating effect was greater at the 1 μM MJ concentration than at 0.1 μM MJ in both cultivars, where the decrease in MDA contents was almost 27% in both the cultivars.

Significant accumulations of H_2_O_2_ and OH^-^ at 200 μM As treatment were observed in the leaves of both cultivars; however, cultivar ZS 758 (black seeded) showed less ROS and suffered less oxidative damage than the cultivar Zheda 622 (yellow seeded sensitive). The H_2_O_2_ content was increased in the leaves of Zheda 622 by 89% and in ZS 758 by 83% when exposed to higher As treatment as compared with the control plants. The addition of exogenous MJ significantly reduced the H_2_O_2_ contents at both MJ concentrations however; 1 μM MJ had better effect (**Figure 2B**) and decreased the H_2_O_2_ contents about 18% in Zheda 622 and 20% in ZS 758. Moreover, OH^-^ contents showed slight difference between the leaves of the control and 50 μM As alone treated plants, but at higher As treatment a significant increase was observed (**Figure 2C**). OH^-^ contents were increased by 30 and 45% respectively in ZS 758 and Zheda 622 as compared with their control plants. Exogenous application of MJ (1 μM) significantly reduced the OH^-^ contents by 17% (ZS 758) and 24% (Zheda 622) in the leaves of *B. napus* plants exposed to higher As concentration (200 μM).

### Antioxidant Enzymes and Gene Expression

**FIGURE 3:**
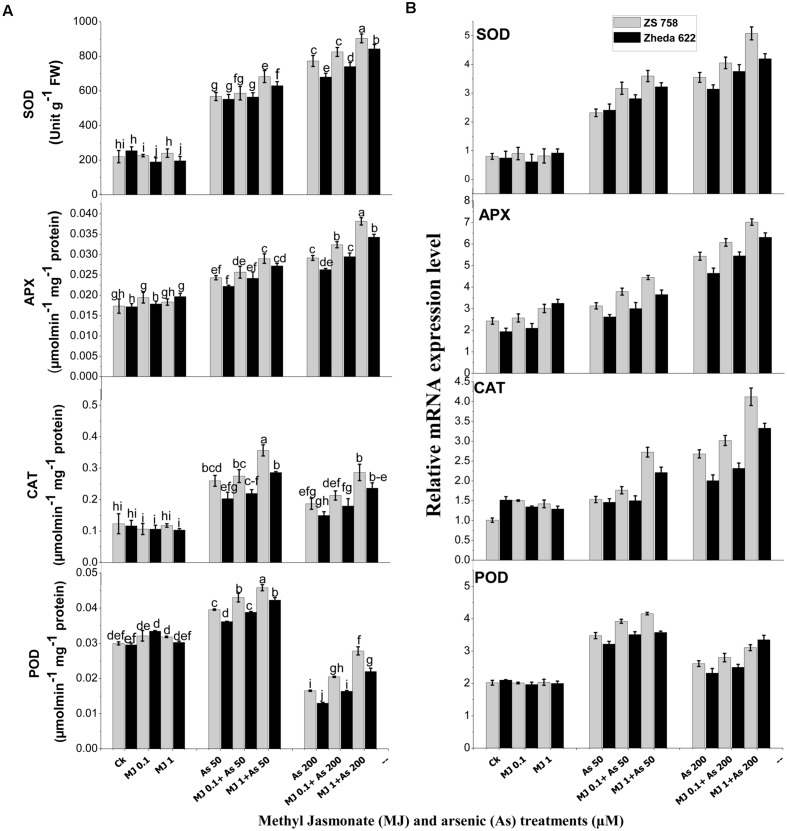
**Effect of different treatments of MJ and arsenic (As) on **(A)** antioxidant enzymes activities and **(B)** related gene expression of superoxide dismutase (SOD), ascorbate peroxidase (APX), catalase (CAT), and peroxidase (POD) in two *B. napus* cultivars.** Vertical bars represent the mean ± SE. Different letters indicate statistically significant differences (*P* ≤ 0.05) by applying Duncan’s multiple range test.

Antioxidant enzyme activities showed significant changes in *B. napus* cultivars when treated with different As levels (**Figure 3A**). The activities of SOD, APX and CAT in the leaves after As treatment (200 μM) increased by 132, 68, and 51% in ZS 758 and 176, 52, and 28% in Zheda 622, respectively as compared with control plants. However, in POD activity a significant decrease was observed in both the cultivars at 200 μM As (**Figure 3A**). The application of MJ further enhanced the activities of antioxidants enzymes compared with untreated control or their respective As treatments. Furthermore, MJ application (1 μM) significantly increased the SOD, APX, CAT, and POD activities by 16, 31, 53, 70% in ZS 758 and 19, 29, 57, and 69% in Zheda 622, respectively at 200 μM As, as compared with As treated plants (**Figure 3A**).

Similarly, qRT-PCR study also confirmed the changes in the activities of *SOD, APX, CAT*, and *POD* against As-induced oxidative stress (**Figure 3B**). The gene expression of *SOD*, *APX*, and *CAT* was found to be induced under As stress, while exogenous MJ application further enhanced the expression of these genes over the respective As treatment (**Figure 3B**). Maximum MJ transcript level was observed in *SOD, APX*, and *CAT* in ZS 758, while the highest *POD* gene expression was observed in Zheda 622 at 1 μM MJ + 200 μM As level (**Figure 3B**).

### Secondary Metabolites and Gene Expression

**FIGURE 4:**
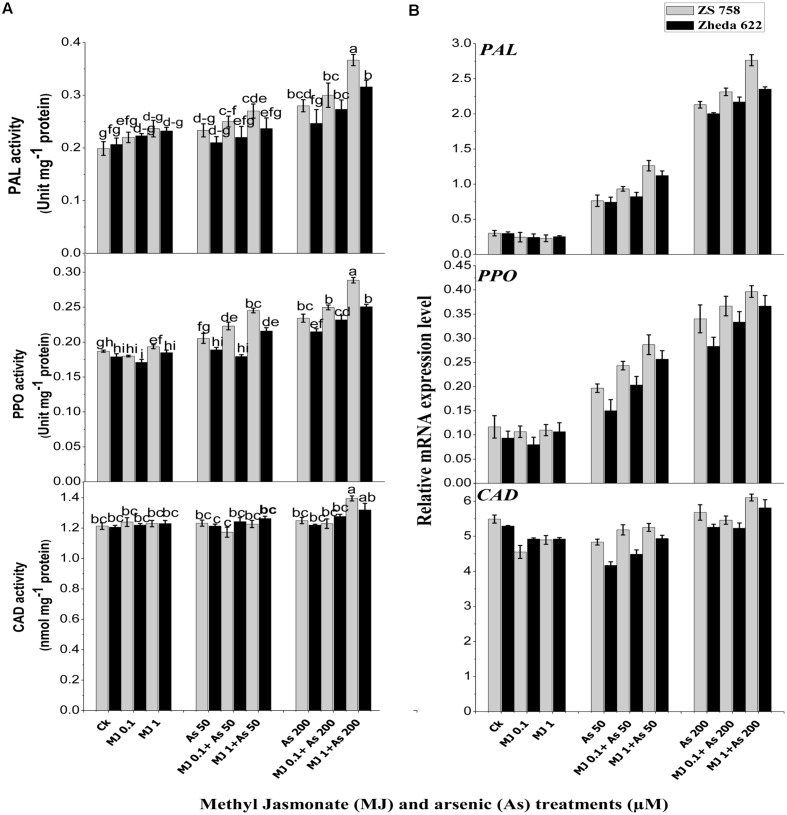
**Effect of different treatments of MJ and arsenic (As) on **(A)** secondary metabolites activities and **(B)** related gene expression of phenylalanine ammonia-lyase (PAL), polyphenol peroxidase (PPO), and cinnamyl alcohol dehydrogenase (CAD) in two *B. napus* cultivars.** Vertical bars represent the mean ± SE. Different letters indicate statistically significant differences (*P* ≤ 0.05) by applying Duncan’s multiple range test.

Analysis of secondary metabolites revealed that activities of PAL and PPO in the leaves of both *B. napus* cultivars were increased and their higher activities were observed at 200 μM As (**Figure 4A**). Exogenous MJ alone had no significant effect, but under As stress it significantly alleviated the As toxicity and further increased the PAL and PPO activities. The highest activity of MJ was observed at 1 μM under As stress, which was 84% in cultivar ZS 758 and 52% in Zheda 622 for PAL. Likewise, PPO activity was increased 54% in ZS 758 and 40% in Zheda 622 over As alone. Exogenously applied MJ also led to a significant increase in *PAL* and *PPO* transcripts compared with As alone treatment (**Figure 4B**). Plants treated with MJ alone did not show any significant changes in *PAL and PPO* transcripts as compared to control. The As treatment (200 μM) induced a small increase in CAD activity and its transcript level in *B. napus* leaves (**Figure 4B**). Although, exogenous MJ and lower As alone treatments did not stimulate the CAD activity and its gene expression, however, MJ application increased the *CAD* transcript level, showing a positive effect of MJ treatment on *CAD* expression. Better effect of MJ was observed at 1 μM under both As stresses. After MJ application (1 μM) the increase in CAD activity under higher As stress was 10% in ZS 758 and 8% in Zheda 622.

### Glutathione Metabolism and Gene Expression

**FIGURE 5:**
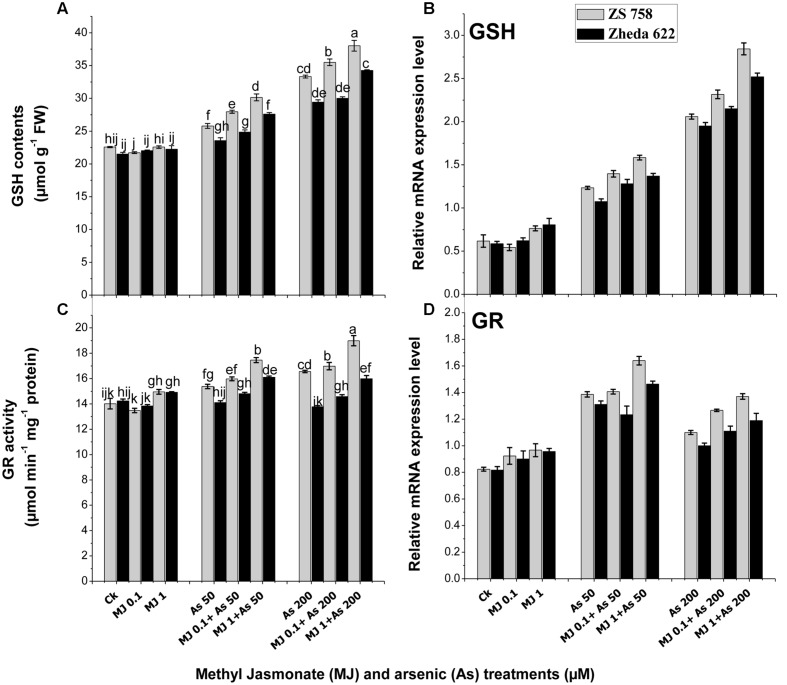
**Effect of different treatments of MJ and arsenic (As) on detoxification-related enzyme activities **(A)** reduced glutathione (GSH) and **(C)** glutathione reductase (GR) and related gene expression of **(B)** GSH and **(D)** GR in two *B. napus* cultivars.** Vertical bars represent the mean ± SE. Different letters indicate statistically significant differences (*P* ≤ 0.05) by applying Duncan’s multiple range test.

The data related with detoxification-related enzymes such as GSH and GR in response to MJ treatments under As stress are presented in **Figure 5.** Under As stress GSH contents increased in both *B. napus* cultivars as compared to control (**Figure 5A**). MJ application to As-stressed plants further improved the GSH content in both *B. napus* cultivars. The highest induction of MJ applications was observed at 1 μM where the increase of GSH content in cultivar ZS 758 was 15% and in Zheda 622 was 13% as compared to As treatment. Similarly, the expression of *GSH* gene under As treatments was also induced as compared to the control plants (**Figure 5B**). Exogenous MJ application further induced the *GSH* transcript level and the highest effect was observed at MJ 1 μM +200 μM As concentration, which was 38% in ZS 758 and 29% in Zheda 622. Moreover, GR activity did not show any significant change in Zheda 622 as compared to control plants (**Figure 5C**). However, MJ application under As stress further enhanced the GR activity and related gene expression in both *B. napus* cultivars as compared to control plants (**Figure 5D**).

### *DHAR, MDHAR* Transcript Level and Ascorbate Contents

**FIGURE 6:**
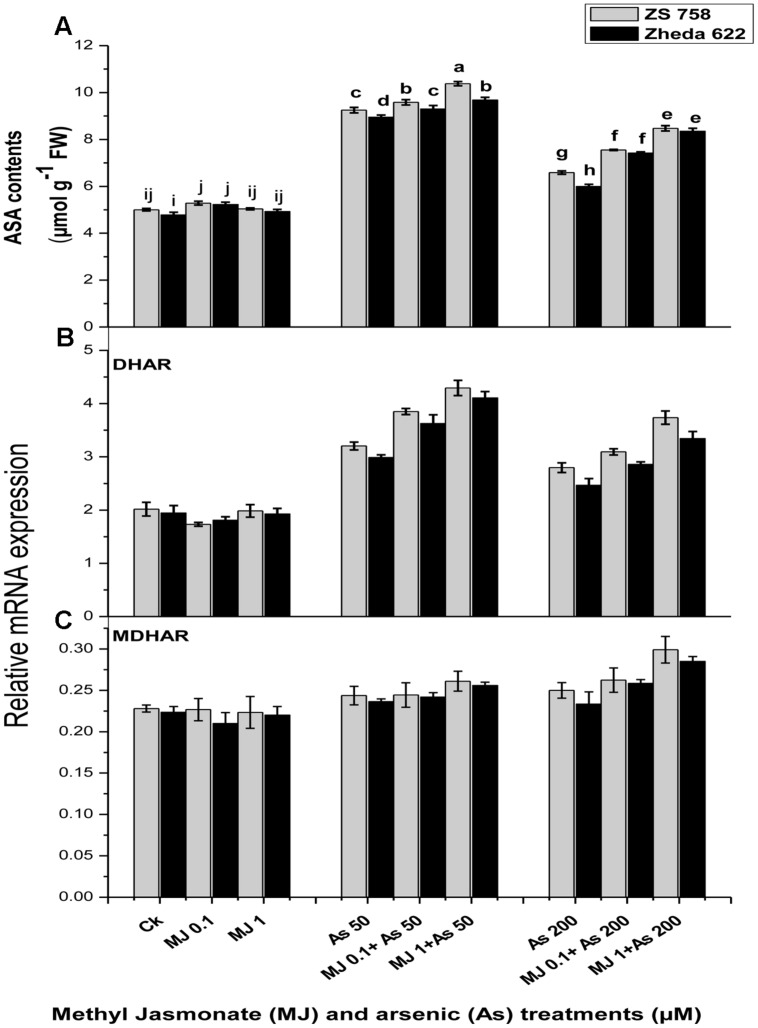
**Effect of different treatments of MJ and arsenic (As) on **(A)** ascorbate contents (ASA) and gene expression of **(B)** dehydroascorbate reductase (*DHAR*) and **(C)** monodehydroascorbate reductase (*MDHAR*) in two *B. napus* cultivars.** Vertical bars represent the mean ± SE. Different letters indicate statistically significant differences (*P* ≤ 0.05) by applying Duncan’s multiple range test.

In order to determine whether the application of MJ could regulate the ascorbate contents under As stress, the effects of MJ treatments on the contents of ascorbate enzyme as well as the recycling pathway related genes such as *DHAR, MDHAR* transcript level were investigated (**Figure 6**). The ASA contents in *B. napus* leaves enhanced after As exposure at 200 μM by 40% in ZS 758 and 33% in Zheda 622. The application of MJ further enhanced the ASA contents over the respective As stressed alone treatment (**Figure 6A**) which was 27% for ZS 758 and 38% for Zheda 622. As stress induced the transcript level of *DHAR* but had no significant effect on *MDHAR* expression (**Figures 6B,C**). The exogenously applied MJ further increased the transcript level of *DHAR* under higher As (200 μM) treatment. However, the transcript level of *MDHAR* slightly increased after 1 μM MJ application under 200 μM As stress, which was more expressed in ZS 758 (**Figure 6C**).

### Endogenous JA Contents and *LOX* Gene Expression

**FIGURE 7:**
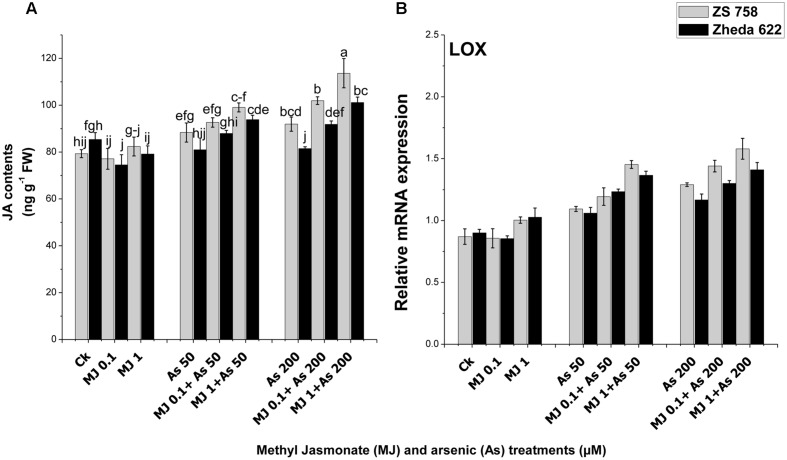
**Effect of different treatments of MJ and arsenic (As) on **(A)** JA contents and gene expression of **(B)** lypoxygenase (*LOX*) in two *B. napus* cultivars.** Vertical bars represent the mean ± SE. Different letters indicate statistically significant differences (*P* ≤ 0.05) by applying Duncan’s multiple range test.

An enhancement in JA content was observed in cultivar ZS 758 after the As treatment (200 μM), while in Zheda 622 the JA content was decreased as compared to their respective controls (**Figure 7A**). Exogenously applied MJ improved JA contents in the leaves of both *B. napus* cultivars, however, significant effect was found at 1 μM MJ (**Figure 7A**). About 14% enhancement in JA contents in cultivar ZS 758 while 10% decrease in Zheda 622 was recorded under As (200 μM) treatment. Exogenous application of MJ increased the JA content about 31% in Zheda 622 and 27% in ZS 758 under the As stress. Furthermore, the analysis of JA synthesis pathway gene *LOX* showed an increase in the expression under the As stress conditions (**Figure 7B**). The addition of MJ in the medium also considerably increased the JA contents in leaves under the higher As level in both the cultivars of *B. napus.*

## Discussion

Heavy metals and metalloids toxicity become most serious concern among abiotic stresses around the world. The stress signals are perceived by several receptors through the transduction to multiple secondary messengers that finally lead the protective responses at the whole-plant level (Sasaki-Sekimoto et al., 2005; Shi and Chan, 2014; Shi et al., 2014). Plants have developed defensive strategies under various environmental stresses that strengthen the plant tolerance even at high levels of potentially toxic compounds (Momoh et al., 2002; Pilon-Smits, 2005). Recently, an extensive work has been carried out on exogenous application of various plant growth regulators to improve plant stress tolerance to different abiotic stresses (Jiang and Zhang, 2001; Horváth et al., 2007; Ali et al., 2015). Similarly, exogenous application of MJ either activates or inhibits plant growth under stress condition depending on its applied concentration and plant species. Piotrowska et al. (2009) found that after 7 or 14 days higher application of JA (100 μM) reduced plant growth and enhanced lead (Pb) accumulation. Whereas, lower dose of JA (0.1 μM) application promoted the plant growth and inhibited the Pb accumulation in *Wolffia arrhiza* L. In most of the studies, it was found that lower MJ concentrations exhibited positive effects on the different metal treated plants (Kim et al., 2009; Jubany-Marí et al., 2010b). For example, the application of MJ at 5 μM showed an ameliorated effect on rice seedlings against cadmium (Cd) stress measured at 3, 7, and 10 days (Singh and Shah, 2014). Similarly, the treatment of low MJ concentration (0.1 μM) enhanced metal tolerance to *Solanum nigrum* L. (Yan et al., 2015) and *Capsicum frutescens* (0.1–1 μM; Yan et al., 2013) under Cd-toxicity at the end of 7 days. Chen et al. (2014) also found that after 9 days of 200 μM Cd treatment, MJ (0.1–1 μM) alleviated the oxidative damage through enhanced antioxidant activities in the *Kandelia obovata* leaves. Furthermore, As concentrations in the present study were based on the findings of our previous experiment (Farooq et al., 2015). While, different concentrations of MJ for the present study were optimized in preliminary experiments (**Table 2**), especially at 200 μM As. In preliminary experiment MJ in different ranges 0.01–50 μM under 200 μM As treatment were used, where 0.1 and 1 μM caused significant increase in plant height but showed non-significant effect on fresh weight on day 3. However, As at 200 μM combined with MJ at 0.1 and 1 μM significantly increased the fresh weight as compared to other MJ treatments on the 8th day, while significant improvement in both fresh weight and plant height were recorded on 14th day. Therefore, MJ applications at 1 μM followed by 0.1 μM were used in this experiment.

**Table 2:** Results of different treatments of MJ under arsenic (As) stress on plant height and fresh weight of two *B. napus* cultivars.

			Three days		Eight days		Fourteen days	
					
	As concentration (μM)	MJ concentration (μM)	Plant height (cm)	Fresh weight (g/plant)	Plant height (cm)	Fresh weight (g/plant)	Plant height (cm)	Fresh weight (g/plant)
ZS 758	200	0	3.5 ± 0.056d	0.08 ± 0.015abc	7.85 ± 0.04c	0.78 ± 0.035cd	12.24 ± 0.05f	1.12 ± 0.025cd
		0.01	3.2 ± 0.035h	0.077 ± 0.003bcd	7.52 ± 0.041e	0.8 ± 0.031bcd	12.28 ± 0.04f	1.08 ± 0.055cde
		0.1	3.77 ± 0.028b	0.09 ± 0.01abc	7.98 ± 0.025b	0.85 ± 0.030b	13.75 ± 0.04b	1.21 ± 0.035b
		1	3.88 ± 0.046a	0.11 ± 0.025a	8.21 ± 0.041a	1.01 ± 0.035a	14.51 ± 0.056a	1.32 ± 0.087a
		10	3.32 ± 0.031f	0.05 ± 0.015d	6.85 ± 0.05g	0.56 ± 0.055g	8.23 ± 0.035i	0.95 ± 0.092fg
		50	3.18 ± 0.048i	0.048 ± 0.002d	6.4 ± 0.028i	0.46 ± 0.04h	7.25 ± 0.04g	0.68 ± 0.026h
Zheda 622	200	0	3.42 ± 0.03e	0.1 ± 0.025ab	7.74 ± 0.04d	0.68 ± 0.051ef	12.52 ± 0.055e	1.02 ± 0.02ef
		0.01	3.29 ± 0.033fg	0.092 ± 0.003ab	7.58 ± 0.026e	0.66 ± 0.04f	11.78 ± 0.02g	1.04 ± 0.03de
		0.1	3.57 ± 0.039d	0.1 ± 0.026a	7 ± 0.039f	0.75 ± 0.039d	12.98 ± 0.03d	1.1 ± 0.087c
		1	3.67 ± 0.025c	0.1 ± 0.04bcd	7.84 ± 0.045c	0.85 ± 0.042bc	13.57 ± 0.045c	1.17 ± 0.042bc
		10	3.25 ± 0.040gh	0.055 ± 0.002cd	6.98 ± 0.049f	0.5 ± 0.055gh	8.85 ± 0.04h	0.91 ± 0.056g
		50	3.32 ± 0.031f	0.06 ± 0.003bcd	6.75 ± 0.055h	0.57 ± 0.045g	6.21 ± 0.045g	0.61 ± 0.035h


*The data are mean ± SE from four replications and different letters within a column show significant difference at *P* ≤ 0.05 according to Duncan’s multiple range test.*

Biomass production was decreased under As stress and this decrease was more obvious under higher As stress (**Table 1**). Further, results showed that MJ causes a significant recovery in the biomass production under As stress. These results proposed that MJ signaling efficiently uncouples the growth from photosynthesis and helps to redirect the biosynthetic capacity from growth to defense (Meldau et al., 2012). Based on proper water and nutrients availability, the rate of plant growth and biomass accumulation is directly associated to the photosynthetic efficiency (Attaran et al., 2014). These results are consistent with the findings of Keramat et al. (2010), who concluded that the biomass production was increased after MJ application in soybean plants under Cd stress. The decrease in photochemical efficiency (Fv/Fm) under the As stress (**Figure 1**) indicated the disturbance in photochemical reactions which may affect the functioning of PSII by blocking electron transport system. Different heavy metals stress has been reported to decrease the chlorophyll fluorescence in various plant species including *B. napus* (Zou et al., 2009; Ali et al., 2015; Kumar and Prasad, 2015). The present results showed that exogenously applied MJ mediated the As-toxicity and improved the Fv/Fm that suggest a positive interaction of MJ to protect the photosynthesis of plants under As stress. Previously, Hristova and Popova (2002) and Zou et al. (2011) reported similar phenomena about the promoting effect of MJ on Fv/Fm, when subjected to stress conditions.

Recently, Yan et al. (2013) reported that MJ applied to *C. frutescens* plants under Cd stress limited the formation of lipid peroxidation as evidenced by reduced content of MDA. Our current results provide a supportive indication that MJ and As treated plants have reduced MDA contents (**Figure 2A**). The application of MJ showed protective effects on the cell membrane lipid and mitigated the As-induced lipid peroxidation in *B. napus*. MJ application in the stressed plant was capable in reducing the MDA contents, which is consistent with previous findings about MJ-induced alleviation of stress due to heavy metals (Singh and Shah, 2014; Hanaka et al., 2016). The overproduction of ROS can provoke partial or severe oxidation resulting in the change of redox state, so the ROS metabolism and their continuous equilibrium is imperative under stress conditions (Jubany-Mari et al., 2010a). The excess formation of ROS under As stress could contribute as a significant constituent and cause damaging effect in the plant cells (Mallick et al., 2011; Finnegan and Weihua, 2012; Farooq et al., 2016). In addition, reduced H_2_O_2_ and OH^-^ contents proved the MJ role in alleviating the As-induced oxidative damage, showing that MJ treatments sustained the H_2_O_2_ and OH^-^ contents in much less concentrations in comparison with the *B. napus* stressed plants. ROS reduction under As stress may be due to the reason that MJ boasts the activities of heme-based molecules and can help in scavenging the ROS under metal toxicity. Oxidative stress alleviation is generally attributed to increased enzyme activities and scavenged ROS formation in the stressful condition (Foyer and Noctor, 2005). Induction of antioxidant defense system prevents the plants to oxidative damage (Sharma et al., 2012). In the present study, different enzyme activities, i.e., SOD, CAT, and APX were induced, while the activity of POD activity was decreased under higher As stress conditions (**Figure 3**). However, addition of MJ acts to maintain the antioxidant defense of *B. napus* plants in adaptation to As stress. It has been reported that MJ provides protection against the tissue decay by increasing the antioxidant enzyme activities and scavenging the free radical (Chanjirakul et al., 2006). The leaves of the yellow seeded *B. napus* cultivar Zheda 622 experienced more oxidative damage due to the overwhelming ROS formation under As stress than those of black seeded cultivar ZS 758, which showed higher antioxidant activities (**Figure 3A**). It has been reported that MJ reduces the metal toxicity by decreasing the oxidative stress in *K. obovata* L. (Chen et al., 2014) and *W. arrhiza* L. (Piotrowska et al., 2009). These findings also clarify that MJ application enhances the enzyme activity (**Figure 3A**) and thus eliminate the oxidative damage through decreasing ROS contents (**Figures 2B,C**). The present study showed that under As stress condition, MJ-treated *B. napus* plants accumulated less ROS and MDA, as compared to As treated plants (**Figure 2**). Our results are in the agreement with those found in *Phaseolus coccineus* where MJ exhibited defensive role in alleviating the stress condition under metal stress (Hanaka et al., 2016). Poonam et al. (2013) also demonstrated that MJ provides self-defense by modulating the antioxidants machinery against the As-induced stress condition and prevents from the oxidative damage. It is worthwhile to note that MJ also enhances enzyme transcript level under As stress as compared to control and subsequently acts to inhibit the As-induced oxidative stress. The increase of MJ-mediated gene expression in As stressed plants showed the triggering effect of MJ to improve metal stress tolerance in *Brassica* plants. In view of these findings, increased levels of these enzyme activities might be the results of *de novo* synthesis or activation of some transcription or translation alterations in specific defense genes in response to MJ application (Santino et al., 2013).

Ascorbic acid and glutathione (GSH) play an essential role in protecting the plants from oxidative stress. ASA is considered as major redox component in plants. In this study, we found that MJ regulated the ascorbate metabolism, as observed by a significant increase in *DHAR* and *MDHAR* transcript levels, as well as the enhanced ASA content under As stress (**Figure 6**). Evidence showed that MJ improved plant stress tolerance by regulating the ascorbate synthesis and recycling the ascorbate accumulation in plants. The increase in ASA by MJ might also be due to the stimulation of MJ responsive genes that encode the ASA biosynthesis, as reported in *Arabidopsis* and tobacco plants (Wolucka et al., 2005). Sasaki-Sekimoto et al. (2005) found ASA accumulation induced by JA under ozone stress in *Arabidopsis* and observed a significant induction of *DHAR* at transcript level. The over expressing of *DHAR* gene in tobacco confirms the resistance to aluminum (Yin et al., 2010) and maintains a high ascorbate level in plants (Yin et al., 2010). Likewise, GSH is another major compound of plant antioxidant system. The GR may reduce the GSSG to GSH and strengthen the detoxification process. Sun et al. (2011) observed that under co-contamination of phenanthrene and As, the GSH contents were increased in *Pteris vittata*. The enhanced GSH redox balance as antioxidative defense influences the cellular signaling pathways responses under stress conditions (Dixit et al., 2011; Foyer and Noctor, 2011). However, similar phenomenon for enhanced GSH content was observed in As-treated *B. napus* plants (**Figure 5**). In *Arabidopsis thaliana* it has been reported that MJ causes accumulation of phytochelatins against Cu and Cd stress (Maksymiec et al., 2007). Dar et al. (2015) reported that MJ might be involved in signaling pathways for GSH metabolic genes and enhanced the synthesis of GSH under metal stress condition. Our present results also showed that MJ could regulate the GSH pathway by inducing the activities and transcript levels of GR and GSH under As stress. On the basis of previous findings of Shan and Liang (2010), it was postulated that MJ functions as signal for the induction of detoxification enzymes and related gene expression under the stressful conditions. They also reported that there was no other signaling pathway for *DHAR* and *MDHAR* genes that activated their transcription except JA. The application of MJ regulated the metabolic pathway of ASA and GSH at transcriptional level and played a major role in tolerance to As-induced stress condition.

In the present study, As stress significantly enhanced the *LOX* expression which was further increased after MJ application. This influence may be arbitrated by MJ, since it has been shown to be a powerful inducer of *LOX* gene expression in other abiotic conditions in different plants such as soybean (Park et al., 1994), *Arabidopsis* (Melan et al., 1993), and potato (Geerts et al., 1994). The expression of *LOX* was increased by exogenous application of MJ and associated with an increased level of endogenous JA (**Figure 7**; Bell and Mullet, 1993; Melan et al., 1993). Results of present study also showed that As treated plants had high contents of JA (**Figure 7**). Increased JA contents under different metal stresses were also observed in *A. thaliana*, *P. coccineus*, rice, and *K. obovata* (Koeduka et al., 2005; Maksymiec et al., 2005; Yan and Tam, 2013). Farmer and Ryan (1990) suggested that JA is an integral part of a general intracellular signal transduction system in response to the stress conditions and regulates inducible defensive genes. Exogenous MJ may be rapidly taken up by the plant cells and hydrolyzed by intracellular esterase to JA and thus may enter the jasmonate signaling pathway (Farmer and Ryan, 1990). Our results showed that MJ treatment significantly increased the JA content in both *B. napus* cultivars (**Figure 7**). Higher level of JA suggests a defensive role in mitigation of excesses ROS during metal stress (Dar et al., 2015). Furthermore, MJ is also involved in the synthesis of secondary metabolites. It was found that treatment of *B. napus* cultivars with As stress caused significant increase in PAL and PPO activities (**Figure 4**). These compounds stimulate the phenylpropanoid pathway and produce phenylpropanoid derivatives (phenols and flavonoids) that play an important role in minimizing the oxidative stress (Wang et al., 2011). Similarly, Ahammed et al. (2013) and Ahmed et al. (2015) also reported that PAL and PPO activities were enhanced under stressed conditions and helped in the removal of ROS contents. In the present study, As-treated plants showed higher activities of PAL and PPO but no induction for CAD activity was observed. However, exogenous application of MJ further regulated the activities of PAL, PPO, and CAD as well as their relative mRNA level (**Figure 4**). Goossens et al. (2003) reported that jasmonate accumulated several secondary metabolites through induction of biosynthetic genes. Thus, it has been proved that JA causes the formation of secondary metabolites and plays a major role in defense response (van der Fits and Memelink, 2000). The induction of these defense genes may assist the plants to cope with the oxidative stress in our study, that is consistent with the finding of Ahammed et al. (2013) who also found that plant growth regulators help in alleviating the oxidative stress in plant leaves through the induction of these defense compounds. Earlier studies have showed that root to shoot metal translocation was significantly reduced under different hormones (Ahammed et al., 2013; Fan et al., 2014). It was found that exogenous MJ significantly increased the phenolic compounds in plants (Kim et al., 2007; Li et al., 2007). It was observed that chamomile plants aerial parts strongly accumulated the phenolic compounds and significantly suppressed the nickel and Cd uptake in shoot (Kováčik et al., 2011). Under such circumstances, there is a possibility that enhanced accumulation of the phenolic compounds in leaves of *B. napus* under MJ application reduced the uptake of As in the leaves. Similar results have also been reported by Piotrowska et al. (2009) and Yan et al. (2015), indicating that exogenous MJ could reduce the Cd and Pb uptake in *S. nigrum* and *W. arrhiza.*

## Conclusion

The results demonstrated that MJ application effectively mitigated the adverse effects of As stress on *B. napus* cultivars and increased the biomass, chlorophyll fluorescence and reduced the MDA and ROS contents. The As stress caused the induction of oxidative stress in the *B. napus* plants due to overproduction of ROS contents. Application of MJ induced the tolerance in *B. napus* plants against As stress by maintaining antioxidant system which plays a key role in scavenging the H_2_O_2_ and OH^-^ contents. The glutathione and ascorbate have important defense responses to oxidative stress and MJ application substantially enhanced the ASA and GSH contents that help in removing the excess ROS. The biosynthesis of secondary metabolites also showed their involvement in tolerance to oxidative stress. The application of MJ significantly reduced the uptake of As and promoted the different plant defense system so that plants were able to cope with the excesses ROS and reduce the effects of oxidative stress. The exogenous application of MJ significantly increased the gene expression of defense compounds under As stress and improved the growth of *B. napus* cultivars. Our results also showed that black seeded cultivar ZS 758 performed better as compared with yellow seeded cultivar Zheda 622 across all the parameters studied. The employment of detailed biological approaches or techniques is needed to determine the precise mechanisms of MJ-induced As stress tolerance in plants.

## Author Contributions

MF and WZ designed the study. RG helped in conducting experiments. MF and FI performed the RNA extractions. BA, HL, SH, and JX analyzed the data and results. MF wrote the manuscript. WZ monitored the experimental work and critically commented on the manuscript. All authors read and approved the final manuscript.

## Conflict of Interest Statement

The authors declare that the research was conducted in the absence of any commercial or financial relationships that could be construed as a potential conflict of interest.
